# Osteopontin is required for full expression of the transformed phenotype by the *ras* oncogene

**DOI:** 10.1054/bjoc.2000.1200

**Published:** 2000-06-15

**Authors:** Y Wu, D T Denhardt, S R Rittling

**Affiliations:** Department of Cell Biology and Neuroscience, Rutgers University, Piscataway, NJ, USA

**Keywords:** tumorigenesis, anchorage-independence, *ras*, integrins, immortalization

## Abstract

The secreted phosphoprotein osteopontin (OPN) is strongly associated with the process of neoplastic transformation, based both on its pattern of expression in vivo and in vitro and on functional analyses. We have used 3T3 cells derived from wildtype and OPN-deficient mice and transformed by transfection with oncogenic *ras* to assess the role of OPN in transformation in vitro and in tumorigenesis in vivo. There was no effect of an absence of OPN on the ability of the cells to undergo immortalization or to form morphologically transformed foci following *ras* transfection. Wildtype and OPN-deficient cell lines were established from such foci, and lines with similar *ras* mRNA levels selected for further analysis. *Ras* -transformed cell lines from both wildtype and OPN-deficient mice could form colonies in soft agar indicating that this process can occur in the absence of OPN. However, the ability of the OPN-deficient cell lines to form colonies was reduced as compared to wildtype cell lines. Tumorigenesis in syngeneic and nude mice was assessed for a subset of cell lines that formed colonies efficiently in soft agar. Cell lines unable to make OPN formed tumors in these mice much more slowly than wildtype cells, despite similar growth of the cells on plastic and in soft agar. Taken together, these results indicate that maximal transformation by *ras* requires OPN expression, and implicate increased OPN expression as an important effector of the transforming activity of the *ras* oncogene. © 2000 Cancer Research Campaign


*British Journal of Cancer* (2000) **83**(2), 156–163
© 2000 Cancer Research Campaign
doi: 10.1054/ bjoc.2000.1200, available online at http://www.idealibrary.com on IDEAL®

### Osteopontin is required for full expression of the transformed phenotype by the *ras* oncogene

**Y Wu, DT Denhardt and SR Rittling[1]**

Department of Cell Biology and Neuroscience, Rutgers University, Piscataway, NJ, USA

**Summary** The secreted phosphoprotein osteopontin (OPN) is strongly associated with the process of neoplastic transformation, based both on its pattern of expression in vivo and in vitro and on functional analyses. We have used 3T3 cells derived from wildtype and OPN-deficient mice and transformed by transfection with oncogenic *ras* to assess the role of OPN in transformation in vitro and in tumorigenesis in vivo. There was no effect of an absence of OPN on the ability of the cells to undergo immortalization or to form morphologically transformed foci following *ras* transfection. Wildtype and OPN-deficient cell lines were established from such foci, and lines with similar *ras* mRNA levels selected for further analysis. *Ras*-transformed cell lines from both wildtype and OPN-deficient mice could form colonies in soft agar indicating that this process can occur in the absence of OPN. However, the ability of the OPN-deficient cell lines to form colonies was reduced as compared to wildtype cell lines. Tumorigenesis in syngeneic and nude mice was assessed for a subset of cell lines that formed colonies efficiently in soft agar. Cell lines unable to make OPN formed tumors in these mice much more slowly than wildtype cells, despite similar growth of the cells on plastic and in soft agar. Taken together, these results indicate that maximal transformation by *ras* requires OPN expression, and implicate increased OPN expression as an important effector of the transforming activity of the *ras* oncogene. © 2000 Cancer Research Campaign

**Keywords:** tumorigenesis, anchorage-independence, *ras*, integrins, immortalization

Changes in cell adhesion properties are hallmarks of the processes of tumorigenesis and metastasis. Indeed, normal cells depend on interaction with the extracellular matrix for growth, and the loss of this dependence is the aspect of cell transformation in vitro most closely correlated with tumorigenicity (Freedman and Shin, 1974). The integrin family of cell surface receptors mediates attachment of cells to the extracellular matrix (reviewed in Hynes, 1992; Juliano, 1994), and alterations in integrin binding or signaling properties are being increasingly recognized as important in tumorigenesis (Ruoslahti, 1997; Clezardin, 1998).

The secreted phosphoprotein osteopontin (OPN), ubiquitously expressed in body fluids, binds with high affinity to integrins of the $\alpha_v$ class (Liaw et al, 1995; Hu et al, 1995*a*; 1995*b*) including $\alpha_v\beta_3$, $\alpha_v\beta_1$, and $\alpha_v\beta_5$. Interaction of the protein with several $\beta_1$ integrins has also been reported recently (Smith et al, 1996; Denda et al, 1998; Bayless et al, 1998), as well as with a non-integrin cell surface receptor, CD-44 (Weber et al, 1996; Katagiri et al, 1999). In vivo, OPN expression is increased in a variety of pathologies (reviewed in Rittling and Denhardt, 1999), where it may act as a macrophage chemoattractant (Singh et al, 1990; Giachelli et al, 1998), or as a repressor of induced nitric oxide synthase (iNOS) levels (Hwang et al, 1994; Rollo et al, 1996): this activity may be especially relevant in pathologies involving ischaemia, in which nitric oxide (NO) production is thought to be an important mediator of tissue damage (Goligorsky et al, 1997; Noiri et al, 1999). In vitro, the protein has cell attachment activity (Somerman et al, 1987; Chambers et al, 1993; Senger et al, 1994), and stimulates migration of several cell types, including endothelial cells (Senger et al, 1996) and smooth muscle cells (Liaw et al, 1995).

There is extensive evidence derived from observations made both in vitro and in vivo linking elevated osteopontin expression and transformation/tumorigenesis. The protein was originally characterized as transformation-associated due to its increased expression in transformed cell lines (Senger et al, 1983): indeed, a wide variety of transformed cells in culture express much higher levels of the protein than their normal counterparts (Senger et al, 1988). These observations have been extended in that among transformed cells, the highest levels of expression were found in the most metastatic cell lines (Chambers et al, 1992*a*). OPN is particularly highly expressed in *ras*-transformed cells (Chambers et al, 1990), and *ras* has a direct effect on OPN transcription, via a *ras*-stimulated transcription factor that enhances transcription of the OPN gene (Guo et al, 1995). OPN is also highly expressed in tumors in vivo. In papillomas and carcinomas induced by DMBA/TPA initiation/promotion in mouse skin, OPN expression levels correlate with tumor stage (Craig et al, 1990). In mammary tumors arising in transgenic mice expressing *ras* and/or *myc* specifically in mammary gland, OPN expression is dramatically increased at both the message and protein level over levels in the corresponding normal mammary gland (Rittling and Novick, 1997). OPN is typically overexpressed in human tumors (Brown et al, 1994; Bellahcène and Castronovo, 1995; Hirota et al, 1995; Tuck et al, 1998), although the source of the protein in these malignancies can be either tumor cells or tumor-associated macrophages (Brown et al, 1994; Casson et al, 1997)

A series of functional studies links OPN causally to tumorigenesis. Experiments in which OPN mRNA level was reduced in

*Received 28 October 1999*
*Accepted 17 February 2000*

*Correspondence to:* SR Rittling already transformed cells indicated that the protein is important in transformation. Thus, transfection of different antisense OPN constructs or ribozymes into transformed, tumorigenic cells resulted in impaired anchorage-independent growth, as well as tumor-forming ability in vivo (Gardner et al, 1994; Su et al, 1995; Feng et al, 1995; Behrend et al, 1994). Even more compelling are results showing that transfection of OPN expression constructs into cells producing benign tumors converts them to a malignant phenotype (Oates et al, 1996; Chen et al, 1997).

In order to more clearly define the role OPN plays in transformation in vitro and tumorigenesis in vivo, we have examined these processes in cells derived from mice with a targeted disruption of the OPN gene (Rittling et al, 1998). The results presented here indicate that while anchorage-independent growth and tumor formation can occur in the absence of OPN, these phenotypes, in particular tumor formation in vivo, are substantially enhanced in wildtype, OPN-expressing cells as compared to OPN deficient ones. Thus, OPN is required for full expression of the transformed phenotype.

### MATERIALS AND METHODS

### Preparation and immortalization of mouse embryo fibroblasts

Mouse embryo fibroblast (MEF) cells were prepared as described (Rittling, 1996). Briefly, mouse embryos were removed from the uterus of 12–16-day pregnant mice, minced finely with scissors and digested with 0.125% trypsin. The cells obtained were plated on 150 mm tissue culture dishes at a density of $1 \times 10^7$ in DMEM (Gibco, Grand Island NY, USA) supplemented with 10% FBS (Gemini Bio-Products, Calabasas, CA, USA). For the first and second passages, cells were plated on 100 mm tissue culture dishes at a density of $1 \times 10^6$; in later passages, cells were plated at 3T3 density ($8 \times 10^5$) on 100 mm dishes. For immortalization, cells were passed every three days and plated at $8 \times 10^5$ on 100 mm dishes (Rittling, 1996). The numbers of cells plated (No), and the numbers of cells after three days of growth (N) were recorded, and the ratio (N/No) was calculated as a growth-rate indicator. Cells were considered to be immortalized when the ratio N/No became higher than 3.0.

### Mice

Wildtype and OPN-deficient mice were housed and bred separately under specific pathogen-free conditions as described (Rittling et al, 1998). Embryos were obtained from homozygous females, either wildtype or OPN-deficient, that had been mated to males of the same genotype. Mice used for embryo collection were in the 129 × C57BL/6 F2 genetic background.

### Tetracycline-inducible cell lines

OPN-deficient 3T3 cells were transfected sequentially with pTet-Off (pUHD-15-1) (Gossen and Bujard, 1992), and with OPN under the control of the Tet operator, and the clones expressing the highest level of OPN selected for further analysis. The resulting clonal lines were transfected with pSV-Ha-*ras*, and foci were picked. Transformed cells from these foci were plated in soft agar at $10^5$ cells per 35 mm well, in the presence or absence of 2 μg $ml^{-1}$ tetracycline.

### Transfection and selection

Plasmid DNA (pSV-Ha-*ras*: 20 μg per 100 mm dish, 10 μg per 60 mm dish) was introduced into cells by the calcium phosphate precipitation method, followed by glycerol shock (Malyankar et al, 1994). Foci, identified as clumps of piled-up cells against a background monolayer, were picked after incubation for 2 weeks with medium containing 3% or 1% FBS, to select for cells with reduced growth factor requirements. Transfection efficiency was assessed by cotransfection with a β-galactosidase expression construct.

### Growth curves

Cells were plated in 12-well dishes at $2.7 \times 10^4$ cells per well. Each day for 6 days, cells were removed from triplicate wells and counted, and the results plotted. Cell number at 3 days after plating, towards the end of the exponential growth phase, is reported.

### Northern and western blotting

RNA preparation, analysis, and probes were as described (Rittling and Novick, 1997). Western blotting for OPN was as in (Rittling et al, 1998*b*) and (Rittling and Feng, 1998), using antiserum 732, developed in the OPN-deficient mice against mouse osteopontin (Kowalski et al, unpublished). For quantification of *ras* protein levels, cells were scraped into RIPA buffer with protein inhibitors, and the protein concentration determined. Equal amounts of protein (25–35 μg) were separated on 12% SDS-PAGE and blotted. The blots were reacted with anti-H-*ras* antibody F235 (Santa Cruz Biotechnology, non-cross reactive with N-*ras* and K-*ras*) at 1 μg $ml^{-1}$.

### Colony formation in soft agar

Anchorage-independent growth assays were performed using 6-well plates (Freedman and Shin, 1974). Experiments were done in triplicate. Each well was coated with 2 ml of a base layer containing 0.6% agar, 1 × DMEM, 10% FBS. Subconfluent cells were collected in 10 ml of medium, counted and suspended in DMEM containing 10% FBS, 0.3% agar and 1 ml of the mixture containing $10^4$ cells was plated over the 2 ml base layer in each well (three wells total for each cell line). After 14 days incubation with twice-a-week feeding (1 ml each time per dish of DMEM, 0.3% agar and 10% FBS), colonies (consisting of about 20 cells or more) in 10 high-power fields per dish were counted, and total colonies per dish calculated.

### Tumor formation

Subconfluent, rapidly growing cells were collected and suspended in PBS at $1 \times 10^6$ cells per ml, and 0.5 ml of this cell suspension was injected subcutaneously in the upper dorsal region of 3–4 syngeneic (129 × C57Bl/6 F1, wildtype) and/or nude mice. Tumor size was measured with calipers every second day until the tumors reached 20% of the weight of the animal. Tumor volume was calculated according to the following formula: $4/3\pi$ (l-1) $(w\text{-}1)^2$, the volume of an oblate ellipse, where l is the long dimension of the tumor, and w is the width, or short dimension, in mm; 1 mm was subtracted from each measurement to compensate for the

© 2000 Cancer Research Campaign 

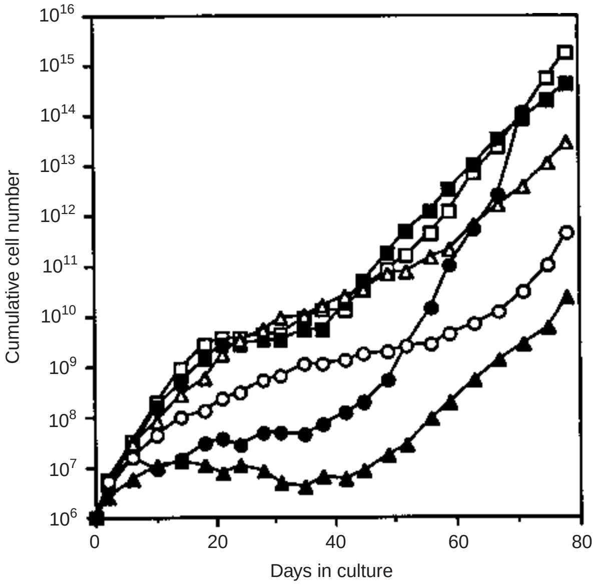

**Figure 1** Immortalization rate of the MEFs derived from wildtype and OPN-deficient mice. Shown is the cumulative cell number in the population plotted as a function of days in culture. Closed symbols represent cells derived from wildtype mice, and the open symbols represent cells derived from the OPN-deficient mice. Each line represents immortalization of cells from a single mouse.

thickness of the skin. In cases where results from only two mice are shown, a third animal in the experiment was sacrificed prior to 20 days, usually because the tumor had grown beyond 20% of the weight of the animal. For metastasis, $10^5$ cells in 0.1 ml of PBS were injected via the lateral tail vein of syngeneic $F_1$ mice, and the mice were sacrificed after 4 weeks. Lungs were excised, fixed and cut into 1–2 mm slices prior to embedding. Several lung slices were embedded in a single block, and tumor size in the resultant H+E stained sections determined by measurement of the projected image. Mice were housed in microisolator cages in an AALAC-approved animal facility, and all procedures were approved by the Rutgers University Institutional Review Board – Animal Care and Facilities Committee.

### RESULTS

### Establishment of immortal and transformed cell lines

Primary embryo fibroblasts were prepared from 13–15-day pregnant females. Initial experiments in which these primary cells were transfected with a mutant *ras* construct together with an activated p53 construct indicated that in both wildtype and OPN-deficient mice, the rate of focus formation was too low to be useful. Therefore, spontaneously immortalized cell lines were derived by continuous passage according to the 3T3 protocol. Three independent mouse embryo fibroblast (MEF) cultures from both wildtype and OPN-deficient mice were obtained, and passed to obtain immortal cell lines. While there is considerable variability in the rate at which different cell lines pass through crisis (Rittling, 1996), there was no consistent difference between the wildtype and OPN-deficient cells (Figure 1), nor was there any consistent difference in the average growth rate of the wildtype and OPN-deficient cell lines (data not shown). Previous work has shown that

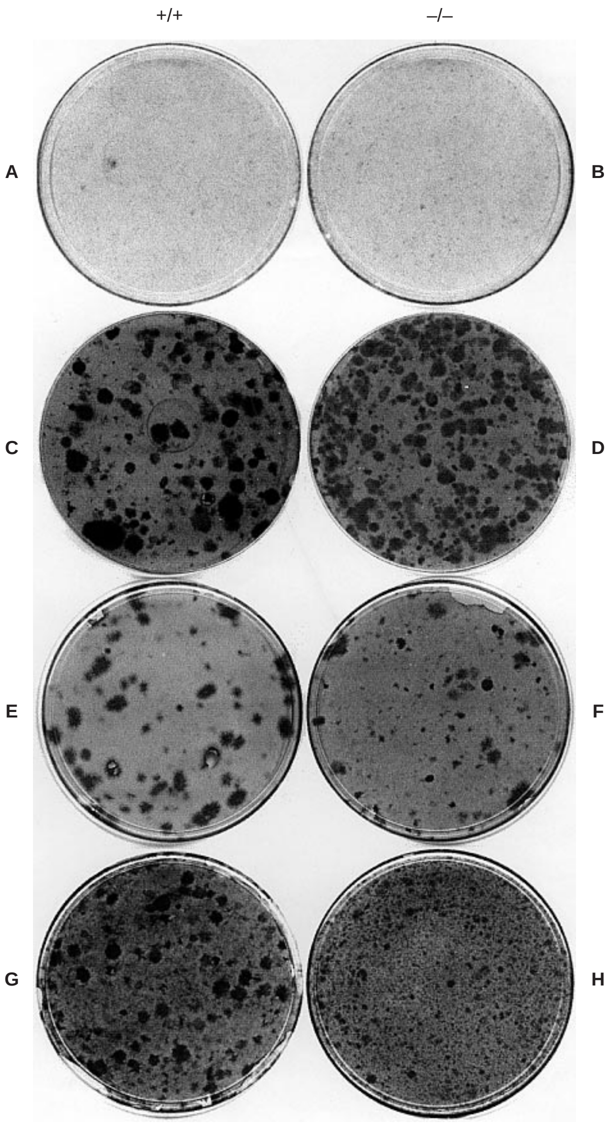

**Figure 2** Comparison of focus-forming ability between *ras*-transfected wildtype and OPN-deficient spontaneously immortalized cells. 3T3 cells were transfected with 20 μg pSV-Ha-*ras* per 100 mm dish using a calcium phosphate precipitation method. After growth at 37°C for 2 weeks in DMEM plus 3% FBS, the cells were fixed with methanol and stained with Giemsa. **A** and **B**: Mock transfection; **A**, **C**, **E**, **G**: wildtype 3T3 lines; **B**, **D**, **F**, **H**: OPN-deficient 3T3 lines. C–H represent focus formation in six independently derived 3T3 lines.

**Table 1** Focus formation in wildtype (+/+) and OPN-deficient (–/–) immortal cell lines after *ras* transfection. 3T3 cell lines were plated at $5 \times 10^5$ cells per 100 mm dish, and transfected with an activated *ras* expression construct. Two weeks following transfection, plates were fixed and stained, and the total foci in one representative 100 mm dish were counted. Transfection efficiency did not vary significantly between the transfections. Results from three independent experiments with six different 3T3 lines are shown.

| Experiment | Number of foci (+/+) | Number of foci (–/–) |
|---|---|---|
| 1 | 84 | 118 |
| 2 | 59 | 67 |
| 3 | 112 | 76 |

© 2000 Cancer Research Campaign

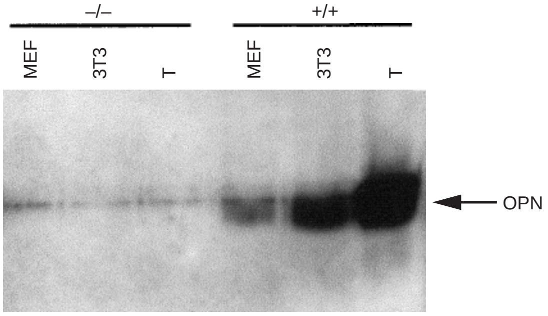

**Figure 3** OPN levels secreted by primary, immortal and transformed cell lines. Media conditioned by confluent cells for 18 h were collected. 10 μl of each medium was used in a western blot with mouse antiserum 732 directed against mouse OPN. A representative set of cells is shown. For each set, medium was collected from primary cells (MEF lanes), immortal cells (3T3 lanes) and transformed cells (T lanes). Similar results were obtained with two other sets of cell lines.

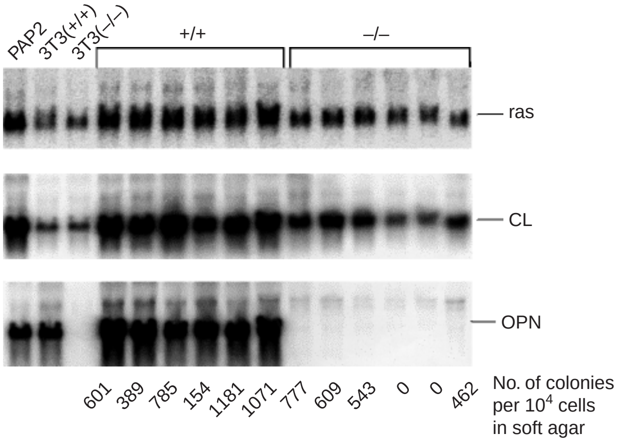

**Figure 4** *ras*, cathepsin L, and OPN mRNA levels and colony formation in soft agar of a series of wildtype (+/+) and OPN-deficient (–/–) transformed cell lines. Cell lines with comparable *ras* levels were selected from the initial screen and expanded. RNA was prepared after 3 days' growth, and growth in soft agar was determined as described in Methods. Lanes labelled PAP2 are RNA isolated from the metastatic, *ras* transformed NIH 3T3 cell line PAP2 (Chambers et al, 1990). Lanes 2 and 3 (3T3) contain RNA prepared from the parental 3T3 cells used for transfection. The same blot was probed for *ras* (top panel), cathepsin L (middle panel), and OPN (lower panel). The experiment shown is representative of three such experiments. The number of colonies formed per $10^4$ cells plated in soft agar for each cell line is shown at the bottom of the figure.

such spontaneously derived immortal cell lines represent fairly homogeneous populations (Rittling, 1996).

These immortal cell lines were transfected with an activated *ras* construct (pSV-Ha-*ras*), and grown in the presence of 3%, or in some cases 1%, fetal bovine serum for 2 weeks, to select for cells with reduced serum requirements. Morphologically transformed foci formed in every case with similar efficiencies (Figure 2, Table 1), and there was no apparent effect of a lack of OPN on focus formation. The average number of foci formed was 85 ± 26.5 for WT cell lines and 87 ± 27.2 for OPN –/– lines.

For each immortal cell line, 10–20 foci were picked, and expanded. RNA was prepared from the resulting transformed cell lines while they were in exponential growth 3 days after plating, and analyzed for *ras* mRNA levels by northern blotting. This screening process was undertaken to select for transformed clones that expressed comparable levels of *ras* mRNA, to minimize differences between clones due to differential expression of the transfected *ras* allele. Thus, in each set of screened clones, wildtype and OPN-deficient clones that expressed similar levels of *ras* mRNA were selected and expanded for further analysis. From six independent immortal cell lines (three of each genotype) a total of 19 wildtype and 17 OPN-deficient *ras*-transformed cell lines were selected for further analysis.

OPN protein levels in the primary, immortal, and transformed cells were monitored by western blotting of media conditioned by the different cells. While the primary wildtype cells secrete readily detectable OPN, the level is increased about 10-fold in the corresponding immortal cells, and increased still further, another sevenfold, after *ras* transfection (Figure 3). No OPN was detected in media conditioned by OPN-deficient cells. While the increase in OPN secretion after *ras* transformation was expected, and confirms that *ras* is active in these transformed cells, these results indicate that immortalization can also increase the amount of OPN secreted by cells.

### Analysis of transformation in vitro

Following selection and expansion of the *ras* transformed cell lines, *ras*, cathepsin L, and OPN mRNA levels were analysed and normalized to β-actin mRNA levels. Cathepsin L expression has been shown in some cases to be, like OPN, induced by activated *ras* (Chambers et al, 1992*b*), and so the level of this mRNA was used as a means of assessing *ras* activity in the individual cell lines. Figure 4 illustrates these data for a single set of cell lines: two additional sets were similarly analysed. Following normalization to β-actin mRNA levels, neither *ras* nor cathepsin L mRNA levels were found to differ significantly between the wildtype and OPN-deficient clones.

The ability of the transformed cell lines to grow under anchorage-independent conditions was tested by seeding $10^4$ cells in 35 mm wells in 0.3% agar in complete medium with 10% FCS. After 2 weeks, colonies containing more than 20 cells were counted. The OPN-deficient cells were able to form colonies in soft agar under these conditions, and in several cell lines colonies were formed with as high efficiency as the wildtype cell lines (Figure 4), and the morphology of the colonies was similar (data not shown). However, when the number of colonies formed per $10^4$ cells plated was averaged over all the cell lines analyzed (19 wildtype and 17 OPN –/–), the wildtype cell lines were found to form nearly twice as many colonies as the OPN-deficient cells (Figure 5), despite considerable variability in the numbers of colonies formed by the individual cell lines. To confirm this observation, *ras* transformed OPN –/– cell lines were developed that expressed OPN under the control of a tetracycline-repressible promoter (Gossen and Bujard, 1992). In these cells OPN expression is reduced about 10-fold in the presence of tetracycline. Growth of these cells in soft agar, but not on plastic, was reduced in the presence of tetracycline by about 20–30% (Figure 5), confirming that OPN under certain circumstances can facilitate anchorage-independent growth of cells.

### Analysis of tumorigenesis in vivo

The ability of transformed cells to form colonies in soft agar is the parameter that most closely correlates with tumor forming ability in vivo (Freedman and Shin, 1974). Accordingly, the ability of several of the wildtype and OPN-deficient cell lines to form tumors after injection subcutaneously into mice was assessed. Four sets of cell lines (wildtype and OPN-deficient) were selected

*© 2000 Cancer Research Campaign*

**Table 2** Growth characteristics and tumour formation of different cell lines with high efficiency of growth in soft agar. The indicated cell lines were injected subcutaneously into mice. At the same passage, the growth rate of the cells on plastic and in soft agar was measured.

| Cell line | Genotype | Growth on plastic $\times 10^{5a}$ | Growth in agar[b] | Tumour size (syngeneic)[c] mm$^3$ | *n* | Tumour size (nude)[c] mm$^3$ | *n* |
|---|---|---|---|---|---|---|---|
| 279–3–7 | +/+ | 4.5 ± 0.1 | 772 ± 64 | 3848,2636 | 2 | 2576 ± 667* | 3 |
| 279–3–12 | +/+ | 3.1 ± 0.4 | 793 ± 110 | 2354,3203 | 2 | 2851 ± 758* | 3 |
| 275–3–2 | +/+ | 6.5 ± 0.5 | 753 ± 70 | 1206 ± 237* | 3 | 2946,5535 | 2 |
| 275–1–4 | +/+ | 1.6 ± 0.2 | 628 ± 35 | 1151 ± 778* | 3 | nd | |
| 247–3–8 | –/– | 2.2 ± 0.0 | 955 ± 203 | 137 ± 106 | 3 | 287 ± 156* | 3 |
| 247–3–12 | –/– | 1.9 ± 0.3 | 1342 ± 239 | 335,264 | 2 | 673 ± 489* | 3 |
| 277–3–4 | –/– | 4.6 ± 0.0 | 810 ± 122 | 46 ± 64* | 3 | 372 ± 253 | 4 |
| 277–1–11 | –/– | 2.8 ± 0.4 | 1502 ± 343 | 44 ± 32* | 3 | nd | |

[a] Growth curves were constructed for each cell line as described in Methods. Total cell number per well at 3 days, when the cells were still in exponential growth, is reported. Numbers represent the average of three determinations ± SD. [b]$10^4$ cells were plated in 0.3% agar, and the total number of colonies present at 14 days determined. Numbers represent the average of three determinations ± SD. [c]$5 \times 10^5$ cells were injected subcutaneously into mice, and tumour size determined after 20 (*) or 21 days. The average total tumour size ± SD for three or four mice is shown, *n* represents the number of mice used. Where $n = 2$, results from individual animals are shown.

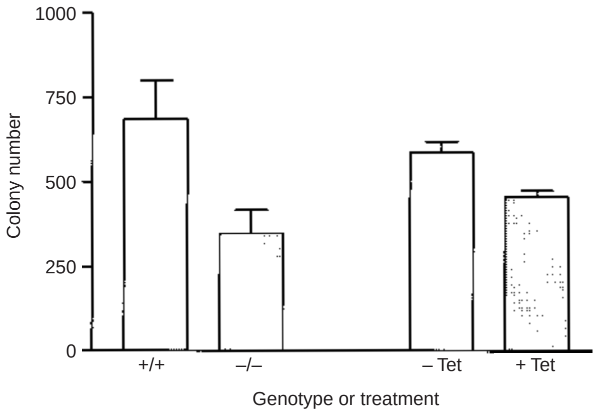

**Figure 5** Colony-forming ability in soft agar of wildtype and OPN-deficient *ras*-transformed cell lines, and in cells with a tetracycline-responsive OPN construct, grown with and without tetracycline. +/+ = average colony formation per $10^4$ cells of 19 wildtype cell lines; –/– = average colony formation per $10^4$ cells of 15 OPN-deficient cells; –Tet = colony formation of $10^5$ OPNTet cells in the absence of Tet (expressing OPN); +Tet = colony formation of $10^5$ OPNTet cells in the presence of Tet (reduced OPN expression). Shown is the mean +/– SEM, $P < 0.05$.

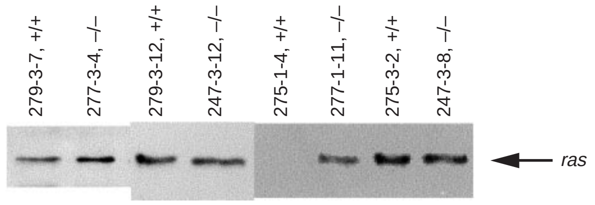

**Figure 6** *ras* protein level in wildtype and OPN-deficient cells used for tumour formation. Cell lysates were prepared from each cell line shown in Table 2, and equal amounts of protein separated on 12% SDS Page gels. *Ras* protein was detected with antibody F235. The designation and genotype of each cell line is shown above the lanes. Cell line 275-1-4 expresses very low levels of *ras*, and may be derived from spontaneously transformed cells.

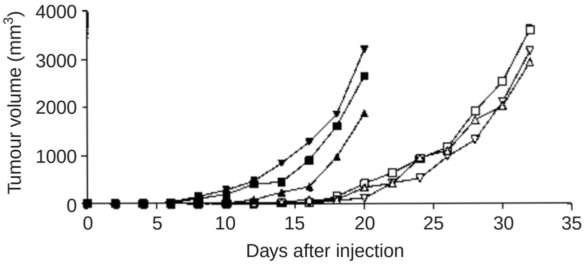

**Figure 7** Growth of tumours in nude mice after injection of wildtype cell line 279-3-7 (solid symbols) or OPN-deficient cell line 247-3-8 (open symbols). $5 \times 10^5$ cells in PBS were injected subcutaneously into the dorsal area of 3–4 mice. Tumour size was measured every other day, and tumour volume calculated as described in Methods. Each line represents results from a single mouse. Results for a representative pair of cell lines are shown – other cell lines gave similar results, as shown in Table 2.

that could form colonies efficiently in soft agar and that had similar *ras* mRNA and protein levels (Figure 6, Table 2). These cells were collected, resuspended in PBS and injected subcutaneously into nude or syngeneic mice. The mice used for these injections were wildtype as there are at present no syngeneic OPN-deficient hosts for these 129 × C57Bl/6 F2 cells. Every second day after the tumors became palpable, the tumor size was measured with calipers. In every case the wildtype cells formed tumors significantly earlier than did the OPN-deficient cell lines (Figure 7) despite comparable growth of the cells in vitro both on plastic and in soft agar (Table 2). Similar results were obtained with both syngeneic and nude mice (Table 2). The main defect observed in the OPN –/– cells was in the lag-time before tumor formation could be detected: once the tumors were formed, the growth rate of the OPN –/– and wildtype tumors was similar (Figure 7). Thus OPN is required for efficient initiation of tumor growth in vivo in this system.

One pair of these cell lines was tested for the ability to form metastases in lungs following intravenous injection into syngeneic mice. The OPN-deficient cells were found to form fewer metastases, (5.5 ± 1.8 metastases per lung section for wt vs 1.2 ± 1.0 for OPN –/– cells; $n = 3$, $P < 0.05$), and those metastases were much smaller than those formed by WT cells (the mean cross-sectional area of the largest metastatic tumor examined in each section was 0.79 ± 0.42 mm$^2$ for wt and 0.162 ± 0.16 mm$^2$ for OPN –/– ($n = 9$; $P < 0.01$)).

© 2000 Cancer Research Campaign

### DISCUSSION

The development of mice deficient for osteopontin expression (Rittling et al, 1998; Liaw et al, 1998) has provided a unique system in which to compare directly the transformed properties of otherwise very similar cells in the presence and absence of osteopontin expression. The work presented here is important in that it provides an uncomplicated analysis of the role of OPN in multiple aspects of cellular transformation. Our results confirm and extend the long-held idea that OPN is important in the transformation process, in that the transformed phenotype was attenuated in the absence of OPN. Thus we observed lower rates on average of colony formation in soft agar, and dramatically reduced rates of primary tumor and metastasis growth in syngeneic and nude mice. The only aspect of transformation for which we did not detect a reduction in the OPN-deficient cells was in the process of focus formation: both the wildtype and osteopontin-deficient cells formed foci efficiently after *ras* transformation. This phenotype, focus formation, most likely represents a loss of contact inhibition of growth resulting from the expression of oncogenic *ras*, and we conclude that OPN, and by deduction also the integrins to which OPN binds in these cells, do not play a major role in this process. We also did not detect an effect of osteopontin on the process of spontaneous immortalization. This process requires an alteration in the cells, probably a mutation (Rittling, 1996) that enables the cells to overcome the growth restraints of senescence.

Our results indicate that the effects of OPN expression on anchorage-independent growth are complex. While antisense experiments suggested that OPN was necessary for the formation of colonies in soft agar (Su et al, 1995; Gardner et al, 1994), we clearly show here that OPN-deficient cell lines can form colonies efficiently in soft agar, indicating that OPN is not absolutely required for this process in *ras*-transformed cells. However, our data show that OPN can enhance anchorage-independent growth. Thus, OPN is not strictly required for anchorage-independent growth, but can enhance such growth in some cell lines. Anchorage-independent growth in *ras*-transformed cells probably results from stimulation by activated *ras* of signaling pathways that mediate both growth factor and integrin-initiated signals (reviewed in Schwartz, 1997). For example, MAPK activation by growth factors in normal cells requires adhesion through integrins (Renshaw et al, 1997), while oncogenic *ras* stimulates MAPK independently of both growth factor and integrin ligation to allow anchorage-independent growth. The binding of soluble OPN to integrins such as the $\alpha_v\beta_3$ may stimulate the signal throughput of these pathways, even in the presence of activated *ras*, thereby enhancing anchorage-independent growth, much as transformed cells that are growth factor independent show higher rates of growth in the presence of growth factors.

The most pronounced effect of a lack of OPN that we observed was in the ability of the OPN-deficient transformed cells to form tumors. For these experiments, a subset of *ras*-transformed cell lines was selected that had similar *ras* levels and formed colonies efficiently in soft agar. While the OPN-deficient clones were able to form tumors in syngeneic or nude mice, they did so at a substantially slower rate than the wildtype cells. Thus, OPN clearly can enhance tumor formation in vivo. It is important to note here that the recipients for these tumor cell injections were wildtype mice, so OPN could have been produced by the stromal cells in the vicinity of the injection of the OPN-deficient cells. Thus host-derived OPN may have contributed to the ultimate ability of the OPN-deficient cells to form tumors. Indeed, we have found (Wu, Feng and Rittling, manuscript in preparation) that tumors arising from the OPN-deficient cells contain measurable amounts of OPN, which may have been supplied by infiltrating macrophages (Brown et al, 1994; Casson et al, 1997) or other sources.

We have considered several hypotheses to explain the mechanism of OPN's ability to enhance tumor formation. It is unlikely that OPN is acting in an autocrine manner to stimulate growth of the tumor cells themselves since these cells already show efficient anchorage-independent growth. Alternatively, high level OPN expression could protect cells from apoptosis in the first few days following tumor cell injection. A role for the OPN-binding integrins $\alpha_5\beta_1$ and $\alpha_v\beta_3$ in preventing apoptosis (or anoikis (Frisch and Ruoslahti, 1997)) of transformed cells has previously been demonstrated (Montgomery et al, 1994; Zhang et al, 1995). Denhardt and Chambers (1994) have proposed that OPN, by virtue of its ability to inhibit iNOS activity, protects tumor cells from attack by cytotoxic cell in vivo, and this may be a possible mechanism of action of OPN in stimulating tumor growth. The possible conflicting role of macrophages in OPN-dependent tumorigenesis has been discussed recently (Crawford et al, 1998). Finally, OPN has been shown to stimulate migration of endothelial cells (Liaw et al, 1995), particularly in association with VEGF (Senger et al, 1996). Possibly, expression of OPN in the injected cells is required for optimal angiogenesis. This idea is supported by the observations that ligation of the $\alpha_v\beta_3$ integrin protects endothelial cells against apoptosis (Brooks et al, 1994*a*; Strömblad et al, 1996) and stimulates angiogenesis (Brooks et al, 1994*b*), and that surface-bound OPN can protect endothelial cells from apoptosis (Scatena et al, 1998). Work is in progress to distinguish these possibilities.

Experiments with transformed cells are hampered by the well-known heterogeneity of such cells, making it difficult to make direct comparisons between different transformed cell lines. Our analysis of a large number of cell lines has allowed us to develop a consensus of the effects of OPN in vitro, despite considerable variability among individual cell lines. The results with tumor formation, however, were less variable, in that every cell line tested showed a slower rate of tumor growth. Thus, it may be that the effects of OPN are more pronounced in the whole animal than in culture, possibly reflecting an important role for this protein in mediating cellular interactions.

### ACKNOWLEDGEMENTS

We wish to thank Aaron Kowalski for help with various aspects of this work, and Dr David Axelrod for critically reviewing the manuscript. Supported by NIH R01 # CA72740 to SRR and NIH grants DC01295 and AR44434 to DTD. Submitted in partial fulfillment of the MSc degree by YW.

### REFERENCES

Bayless KA, Meininger GA, Scholtz JM and Davis GE (1998) Osteopontin is a ligand for the $\alpha_4\beta_1$ integrin. *J Cell Sci* **111**: 1165–1174

Behrend EI, Craig AM, Wilson SM, Denhardt DT and Chambers AF (1994) Reduced malignancy of *ras*-transformed NIH 3T3 cells expressing antisense osteopontin RNA. *Cancer Res* **54**: 832–837

Bellahcène A and Castronovo V (1995) Increased expression of osteonectin and osteopontin, two bone matrix proteins, in human breast cancer. *Am J Pathol* **146**: 95–100

Brooks PC, Clark RAF and Cheresh DA (1994*a*) Requirement of vascular integrin $\alpha_v\beta_3$ for angiogenesis. *Science* **264**: 569–571

© 2000 Cancer Research Campaign

Brooks PC, Montgomery AM, Rosenfeld M, Reisfeld RA, Hu T, Klier G and Cheresh DA (1994*b*) Integrin $\alpha_v\beta_3$ antagonists promote 1 regression by inducing apoptosis of angiogenic blood vessels. *Cell* **79**: 1157–1164

Brown LF, Papadopoulos-Sergiou A, Berse B, Manseau EJ, Tognazzi K, Perruzzi CA, Dvorak HF and Senger DR (1994) Osteopontin expression and distribution in human carcinomas. *Am J Pathol* **145**: 610–623

Casson AG, Wilson SM, McCart JA, O'Malley FP, Ozcelik H, Tsao MS and Chambers AF (1997) *ras* mutation and expression of the *ras*-regulated genes osteopontin and cathepsin L in human esophageal cancer. *Int J Cancer* **72**: 739–745

Chambers AF, Denhardt DT and Wilson SM (1990) RAS-transformed NIH3T3 cell lines, selected for metastatic ability in chick embryos, have increased proportions of p21-expressing cells and are metastatic in nude mice. *Invasion Metastasis* **10**: 225–240

Chambers AF, Behrend EI, Wilson SM and Denhardt DT (1992*a*) Induction of expression of osteopontin (OPN, secreted phosphoprotein) in metastatic, *ras*-transformed NIH-3T3 cells. *Anticancer Res* **12**: 43–48

Chambers AF, Colella R, Denhardt DT and Wilson SM (1992*b*) Increased expression of cathepsins L and B and decreased activity of their inhibitors in metastatic, *ras*-transformed NIH 3T3 cells. *Mol Carcinog* **5**: 238–245

Chambers AF, Hota C and Prince CW (1993) Adhesion of metastatic, *ras*-transformed NIH 3T3 cells to osteopontin, fibronectin, and laminin. *Cancer Res* **53**: 701–706

Chen H, Ke Y, Oates AJ, Barraclough R and Rudland PS (1997) Isolation of and effector for metastasis-inducing DNAs from a human metastatic carcinoma cell line. *Oncogene* **14**: 1581–1588

Clezardin P (1998) Recent insights into the role of integrins in cancer metastasis. *Cell Mol Life Sci* **54**: 541–548

Craig AM, Bowden GT, Chambers AF, Spearman MA, Greenberg AH, Wright JA, McLeod M and Denhardt DT (1990) Secreted phosphoprotein mRNA is induced during multistage carcinogenesis in skin and correlates with the metastatic potential of murine fibroblasts. *Int J Cancer* **46**: 133–137

Crawford HC, Matrisian LM and Liaw L (1998) Distinct roles of osteopontin in host defense activity and tumour survival during squamous cell carcinoma progression in vivo. *Cancer Res* **58**: 5206–5215

Denda S, Reichardt LF and Müller U (1998) Identification of osteopontin as a novel ligand for the integrin $\alpha_8\beta_1$ and potential roles for this integrin-ligand interaction in kidney morphogenesis. *Mol Biol Cell* **9**: 1425–1435

Denhardt DT and Chambers AF (1994) Overcoming obstacles to metastasis – a defense against host defenses: Osteopontin (OPN) as a shield against attack by cytotoxic host cells. *J Cell Biochem* **56**: 48–51

Feng B, Rollo EE and Denhardt DT (1995) Osteopontin (OPN) may facilitate metastasis by protecting cells from macrophage NO-mediated cytotoxicity: evidence from cell lines down-regulated for OPN expression. *Clin Exp Metastasis* **13**: 453–462

Freedman VH and Shin SI (1974) Cellular tumourigenicity in nude mice: correlation with cell growth in semi-solid medium. *Cell* **3**: 355–359

Frisch SM and Ruoslahti E (1997) Integrins and anoikis. *Curr Opin Cell Biol* **9**: 701–706

Gardner HAR, Berse B and Senger DR (1994) Specific reduction in osteopontin synthesis by antisense RNA inhibits the tumourigenicity of transformed Rat1 fibroblasts. *Oncogene* **9**: 2321–2326

Giachelli CM, Lombardi D, Johnson RJ, Murry CE and Almeida M (1998) Evidence for a role of osteopontin in macrophage infiltration in response to pathological stimuli *in vivo*. *Am J Pathol* **152**: 353–358

Goligorsky MS, Noiri E, Kessler H and Romanov V (1997) Therapeutic potential of RGD peptides in acute renal injury. *Kidney Int* **51**: 1487–1492

Gossen M and Bujard H (1992) Tight control of gene expression in mammalian cells by tetracycline-responsive promoters. *Proc Natl Acad Sci USA* **89**: 5547–5551

Guo X, Zhang YP, Mitchell DA, Denhardt DT and Chambers AF (1995) Identification of a *ras*-activated enhancer in the mouse osteopontin promoter and its interaction with a putative ETS-related transcription factor whose activity correlates with the metastatic potential of the cell. *Mol Cell Biol* **15**: 476–487

Hirota S, Ito A, Nagoshi J, Takeda M, Kurata A, Takatsuka Y, Kohri K, Nomura S and Kitamura Y (1995) Expression of bone matrix messenger ribonucleic acids in human breast cancers. Possible involvement of osteopontin in development of calcifying foci. *Lab Invest* **72**: 64–69

Hu DD, Hoyer JR and Smith JW (1995*a*) Calcium suppresses cell adhesion to osteopontin by attenuating binding affinity for integrin $\alpha_v\beta_3$. *J Biol Chem* **270**: 9917–9925

Hu DD, Lin ECK, Kovach NL, Hoyer JR and Smith JW (1995*b*) A biochemical characterization of the binding of osteopontin to integrins $\alpha_v\beta_1$ and $\alpha_v\beta_5$. *J Biol Chem* **270**: 26232–26238

Hwang SM, Lopez CA, Heck DE, Gardner CR, Laskin DL, Laskin JD and Denhardt DT (1994) Osteopontin inhibits induction of nitric oxide synthase gene expression by inflammatory mediators in mouse kidney epithelial cells. *J Biol Chem* **269**: 711–715

Hynes RO (1992) Integrins: versatility, modulation, and signaling in cell adhesion. *Cell* **69**: 11–25

Juliano RL (1994) Signal transduction by integrins and its role in the regulation of tumour growth. *Cancer Metastasis Rev* **13**: 25–30

Katagiri YU, Sleeman J, Fujii H, Herrlich P, Hotta H, Tanaka K, Chikuma S, Yagita H, Okumura K, Murakami M, Saiki I, Chambers AF and Uede T (1999) CD-44 variants but not CD-44s cooperate with beta-1 containing integrins to permit cells to bind to osteopontin independently of arginine-glycine-aspartic acid, thereby stimulating cell motility and chemotaxis. *Cancer Res* **59**: 219–226

Liaw L, Skinner MP, Raines EW, Ross R, Cheresh DA, Schwartz SM and Giachelli CM (1995) The adhesive and migratory effects of osteopontin are mediated via distinct cell surface integrins: role of $\alpha_v\beta_3$ in smooth muscle migration to osteopontin *in vitro*. *J Clin Invest* **95**: 713–724

Liaw L, Birk DE, Ballas CB, Whitsitt JS, Davidson JM and Hogan BLM (1998) Altered wound healing in mice lacking a functional osteopontin gene (spp1). *J Clin Invest* **101**: 1468–1478

Malyankar UM, Rittling SR, Connor A and Denhardt DT (1994) The mitogen-regulated protein/proliferin transcript is degraded in primary mouse embryo fibroblast but not 3T3 nuclei: altered RNA processing correlates with immortalization. *Proc Natl Acad Sci USA* **91**: 335–339

Montgomery AM, Reisfeld RA and Cheresh DA (1994) Integrin alpha v beta 3 rescues melanoma cells from apoptosis in three- dimensional dermal collagen. *Proc Natl Acad Sci USA* **91**: 8856–8860

Noiri E, Dickman K, Miller F, Romanov G, Romanov V, Shaw R, Rittling SR, Denhardt DT and Goligorsky MS (1999) Acute renal ischemia in mice with a targeted disruption of the osteopontin gene: link to nitric oxide-induced injury. *Kidney Int* **56**: 74–83

Oates AJ, Barraclough R and Rudland PS (1996) The identification of osteopontin as a metastasis-related gene product in a rodent mammary tumour model. *Oncogene* **13**: 97–104

Renshaw MW, Ren XD and Schwartz MA (1997) Growth factor activation of MAP kinase requires cell adhesion. *EMBO J* **18**: 5592–5599

Rittling SR (1996) Clonal nature of spontaneously immortalized 3T3 cells. *Exp Cell Res* **229**: 7–13

Rittling SR and Novick KW (1997) Osteopontin expression in mammary gland development and tumorigenesis. *Cell Growth Differ* **8**: 1061–1069

Rittling SR and Feng F (1998) Detection of mouse osteopontin by western blotting. *Biochem Biophys Res Commun* **250**: 287–292

Rittling SR and Denhardt DT (1999) Osteopontin (OPN) function in pathology: lessons from OPN-deficient mice. *Experimental Nephrology* **7**: 103–113

Rittling SR, Matsumoto HN, McKee MD, Nanci A, An XR, Novick KE, Kowalski AJ, Noda M and Denhardt DT (1998) Mice lacking osteopontin show normal development and bone structure but display altered osteoclast formation *in vitro*. *J Bone Miner Res* **13**: 1101–1111

Rollo EE, Laskin DL and Denhardt DT (1996) Osteopontin inhibits nitric oxide production and cytotoxicity by activated RAW 264.7 macrophages. *J Leukoc Biol* **60**: 397–404

Ruoslahti E (1997) Integrins as signaling molecules and targets for tumour therapy. *Kidney Int* **51**: 1413–1417

Scatena M, Almeida M, Chaisson ML, Fausto N, Nicosia RF and Giachelli CM (1998) NF-κB mediates $\alpha_v\beta_3$ integrin-induced endothelial cell survival. *J Cell Biol* **141**: 1083–1093

Schwartz MA (1997) Integrins, oncogenes and anchorage independence. *J Cell Biol* **139**: 575–578

Senger DR, Asch BB, Smith BD, Perruzzi CA and Dvorak HF (1983) A secreted phosphoprotein marker for neoplastic transformation of both epithelial and fibroblastic cells. *Nature* **302**: 714–715

Senger DR, Perruzzi CA, Gracey CF, Papadopoulous A and Tenen DG (1988) Secreted phospohoproteins associated with neoplastic transformation: close homology with plasma proteins cleaved during blood coagulation. *Cancer Res* **48**: 5770–5774

Senger DR, Perruzzi CA, Papadopoulous-Sergiou A and Van De Water L (1994) Adhesive properties of osteopontin: regulation by a naturally occurring thrombin-cleavage in close proximity to the GRGDS cell-binding domain. *Mol Biol Cell* **5**: 565–574

Senger DR, Ledbetter SR, Claffey KP, Papadopoulous-Sergiou A, Perruzzi CA and Detmar M (1996) Stimulation of endothelial cell migration by vascular permeability factor/vascular endothelial growth factor through cooperative mechanisms involving the $\alpha_v\beta_3$ integrin, osteopontin, and thrombin. *Am J Pathol* **149**: 293–305

*© 2000 Cancer Research Campaign*

Singh RP, Patarca R, Schwartz J, Singh P and Cantor H (1990) Definition of a specific interaction between the early T lymphocyte activation 1 (Eta-1) protein and murine macrophages in vitro and its effect upon macrophages in vivo. *J Exp Med* **171**: 1931–1942

Smith LL, Cheung HK, Ling LE, Chen J, Sheppard D, Pytela R and Giachelli CM (1996) Osteopontin N-terminal domain contains a cryptic adhesive sequence recognized by $\alpha_9\beta_1$ integrin. *J Biol Chem* **271**: 28485–28491

Somerman MJ, Prince CW, Sauk JJ, Foster RA and Butler WT (1987) Cell attachment activity of the 44 kilodalton bone phosphoprotein is not restricted to bone cells. *Matrix* **9**: 49–54

Strömblad S, Becker JC, Yebra M, Brooks PC and Cheresh DA (1996) Suppression of p53 activity and $p21^{WAF1/CIP1}$ expression by vascular cell integrin $\alpha_v\beta_3$ during angiogenesis. *J Clin Invest* **98**: 426–433

Su L, Mukherjee AB and Mukherjee BB (1995) Expression of antisense osteopontin mRNA inhibits tumour promoter-induced neoplastic transformation of mouse JB6 epidermal cells. *Oncogene* **10**: 2163–2169

Tuck AB, O'Malley FP, Singhal H, Tonkin KS, Kerkvliet N, Saad Z, Doig GS and Chambers AF (1998) Osteopontin expression in a group of lymph node negative breast cancer patients. *Int J Cancer* **79**: 502–508

Weber GF, Ashkar S, Glimcher MJ and Cantor H (1996) Receptor-ligand interaction between CD44 and osteopontin (ETA-1). *Science* **271**: 509–512

Zhang Z, Vuori K, Reed JC and Ruoslahti E (1995) The $\alpha_5\beta_1$ integrin supports survival of cells on fibronectin and up-regulates Bcl-2 expression. *Proc Natl Acad Sci USA* **92**: 6161–6165

*© 2000 Cancer Research Campaign*
